# Flux sampling and graph neural networks for improved gene essentiality prediction in mammalian genome-scale metabolic models

**DOI:** 10.1038/s41540-026-00738-8

**Published:** 2026-05-08

**Authors:** Kieren Sharma, Lucia Marucci, Zahraa S. Abdallah

**Affiliations:** School of Engineering Mathematics and Technology, University of Bristol, Bristol, UK

**Keywords:** Computational biology and bioinformatics, Mathematics and computing, Systems biology

## Abstract

Gene essentiality, the requirement of a gene for survival or proliferation, is central to understanding cellular processes and identifying drug targets. Experimental determination requires large growth screens that are time-consuming and expensive, motivating in silico approaches. Existing methods predominantly use flux balance analysis (FBA), a constraint-based optimisation framework that requires a predefined cellular objective function. This can introduce observer bias, because the objective often reflects the researcher’s assumptions rather than the cell’s biological goals. Here, we present FluxGAT, a graph neural network (GNN) that predicts gene essentiality from graphical representations of flux sampling data. Flux sampling removes the need for an explicit objective and instead characterises feasible steady-state fluxes. FluxGAT combines this information with metabolic network topology to learn flux-informed node representations and classify reactions as essential or non-essential. We apply FluxGAT to the iCHO2291 genome-scale model of Chinese hamster ovary cells and Mouse1, a generic mouse model with independent essentiality labels. In both systems, FluxGAT improves sensitivity over FBA while maintaining high precision and specificity, and recovers more experimentally essential genes, especially where FBA predicts very few essentials. These results show that flux-informed GNNs can provide more general gene essentiality predictions across mammalian genome-scale models without hand-crafted objective functions.

## Introduction

Gene essentiality, defined by the necessity of specific genes for cellular reproduction^1^, is critical for understanding the basic requirements of a cell^2^, accelerating drug target discovery^3–7^, and guiding the engineering of organisms for chemical production^8^. While advancements in genome sequencing and CRISPR-based genome editing have improved our ability to identify essential genes, experimental methods remain time-consuming, costly, and not always feasible across all organisms. This has led to a shift towards in silico approaches for predicting gene essentiality, which analyse intrinsic genomic features, topological features of biological networks^9^, and combinations of features using machine learning (ML)^10^.

ML approaches involve training classifiers using known *essential* and *non-essential* genes, integrating features such as gene and protein sequences, network topology, gene ontology, and homology^9–11^. While traditional ML methods like decision trees (DTs) and support vector machines (SVMs) are commonly used, deep learning neural networks are less frequent due to challenges with interpretability of ‘black-box’ models^12^.

Building on these feature-based approaches, network-based methods infer essentiality from how genes or proteins are connected within biological interaction graphs. In practice, they use node-level topological properties in networks such as protein-protein interaction networks (PPIs), so predictions can be made from interaction structure even when sequence-derived features are limited or no new sequencing experiments are available. Graph neural networks (GNNs), designed for graph-structured data^13^, are well suited to tasks that require an understanding of network relationships and are likely to become central to single-cell analysis within biomedicine for this reason^14^.

In metabolic engineering, an expanding area of synthetic biology, flux balance analysis (FBA) is a popular mathematical approach for studying cellular phenotypes and is commonly used to predict gene essentiality. FBA utilises constraint-based modelling and linear programming to predict the distribution of metabolites across a metabolic network under steady-state conditions, assuming a predefined cellular fitness objective^15^. However, defining a cellular objective introduces observer bias, because the chosen objective may not fully capture the actual biological goals of an organism^16–19^, which are dynamic and environment dependent.

Flux sampling addresses this limitation by exploring a range of possible flux states without requiring a predefined objective function^20^. Sampling algorithms repeatedly draw feasible flux states to cover the entire solution space, yielding probability distributions of steady-state reaction fluxes for downstream analysis. Flux sampling has shown improved performance over FBA in predicting flux distributions^21,22^, particularly in complex mammalian cell lines where a single objective function may not represent natural conditions^21^.

Recent work has begun to combine ML with genome-scale metabolic models (GSMMs) for metabolic gene essentiality prediction. ML-based methods have demonstrated comparable performance to FBA for predicting metabolic gene essentiality, using only wild-type flux distributions where cellular objectives are more likely to hold^11^. By only applying FBA to the wild-type cell, they eliminate the need to assume that deletion strains optimise the same fitness objective as the wild type. Their work utilised four traditional ML classifiers to leverage manually extracted features from a graph-structured representation of metabolic fluxes, where nodes represent enzymatic reactions, and edges quantify the mass flow of metabolites between these reactions. Although the model could capture most of the essential genes determined experimentally, there was a high rate of false positive predictions.

This line of work was recently extended by employing a GNN framework for increased predictive power over conventional ML methods for classifying essentiality using the same graphical representation^23^. Comparative analysis of their model’s predictions with FBA reveals a performance comparable to FBA in identifying essential genes. However, it is noteworthy that FBA exhibits significantly superior performance in identifying non-essential genes. The significance of their results lies in the generalisation ability of the model to accurately predict gene essentiality across various growth conditions. A critical limitation, however, is the reliance on the assumption of a wild-type fitness objective, which restricts the model’s applicability to model organisms where such behaviour is well-documented. This work motivates the analysis of enzymatic reaction networks in predicting gene essentiality.

Beyond GSMM-based approaches, a GNN model was employed to predict gene essentiality using PPI network data^24^, significantly outperforming traditional ML and network-based methods. However, challenges arise due to the inherent noise and the prevalence of false positive connections in PPI networks. Additionally, the existence of multiple protein interaction databases, each presenting considerably varied network structures, can lead to inconsistent and unstable outcomes. This highlights the importance of GSMMs as highly utilised tools in systems biology, offering more robust frameworks for studying cellular phenotypes.

To our knowledge, there is currently no existing work, utilising ML or other methodologies, that focuses on analysing GSMM flux sampling data for the specific aim of predicting gene essentiality. Consequently, this limits the direct comparability of our approach to existing methods, making FBA the primary benchmark for evaluating performance.

**Fig. 1:**
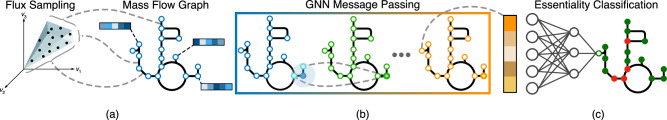
Overview of the FluxGAT architecture for predicting reaction essentiality from flux sampling data. **a** Flux sampling is used to construct a weighted graph of a metabolic reaction network. **b** The adjacency matrix, along with node features containing chemical properties of reactions, are then passed through an optimised graph attention network (GAT) which iteratively generates new representations for each reaction using neural message-passing layers. **c** Lastly, a dense neural layer and sigmoid activation function are used to perform binary classification from each node’s final embedding, learning to predict if an enzymatic reaction is essential (red) or non-essential (green) for cell growth.

In this work, we introduce FluxGAT (Fig. 1), a GNN-based approach for metabolic gene essentiality prediction that learns representations from flux sampling data. We first apply FluxGAT to the most complete genome-scale metabolic model (GSMM) of Chinese hamster ovary (CHO) cells, iCHO2291^25^, which are the most commonly used mammalian hosts in the biopharmaceutical industry^26^. We then evaluate the model on Mouse1, a generic mouse GSMM with independent essentiality labels, to assess whether the approach generalises across mammalian systems. By using flux sampling rather than an explicit objective function, FluxGAT reduces the observer bias present in traditional FBA-based methods. This is important for mammalian and non-model organisms, where objectives are not well defined and existing methods therefore fall short. We first evaluate FluxGAT on iCHO2291 and compare it with both conventional ML classifiers and FBA, then test whether the same workflow generalises to Mouse1. To isolate the contribution of reaction flux information from network topology, we run an ablation that removes flux-derived edge weights while keeping the directed graph structure intact. This shows that most of the predictive signal comes from the active graph topology, with edge-weights providing a smaller additional improvement. Full methodological details on flux sampling, graph construction, label mapping and model training are provided in the ‘Methods’.

## Results

We first applied the artificial centring hit-and-run (ACHR) flux sampling algorithm to the wild-type iCHO2291 GSMM to characterise feasible steady-state flux behaviour, from which we constructed a mass flow graph (MFG) in which nodes represent enzymatic reactions and directed weighted edges encode metabolite flow between reactions^27^. Experimental CHO gene essentiality labels were then mapped onto reaction nodes through the gene-protein-reaction (GPR) rules, and each reaction was represented by features encoding its associated reactants and products. This defined a reaction-level binary node-classification task on a weighted directed graph, allowing FluxGAT to learn from both network connectivity and reaction chemistry when predicting essential and non-essential nodes. We then compared its performance with traditional classifiers and FBA, before extending the same workflow to Mouse1.

### Dataset

The ACHR algorithm was first applied to the wild-type iCHO2291 GSMM of CHO cells^25^, which contains *m* = 3972 metabolites and *n* = 6236 reactions. Sampling was performed for 50,000 iterations with a *thinning* value of 1000 to reduce the memory footprint of the sampling results whilst still ensuring meaningful convergence^28^. Computational resources, software libraries, approximate runtimes, and code availability are described in the Supplementary Information. Averaging the probability distributions for each reaction resulted in a single flux vector $\overline{{\bf{v}}}$ with 5316 non-zero reaction fluxes. From the sampled mean flux vector, we constructed a MFG, a directed reaction-level graph in which edges encode weighted metabolite flow between reactions, containing 4733 nodes. In this representation, reversible reactions can be unfolded into separate forward and reverse nodes when they carry feasible flux, so the number of MFG nodes need not match the number of reactions in the underlying GSMM. A schematic of its topology is provided in Supplementary Fig. S3. The MFG also contains 335,024 weighted and directed edges, representing the directionality and strength of metabolite flow between reactions.

Because the MFG is defined over reactions rather than genes, experimental gene essentiality labels were mapped onto reaction nodes through the model’s GPR rules. Function *f* (Eq. (8)) was applied to generate these binary essentiality labels for nodes in the MFG using experimental gene essentiality values taken from a genome-wide CRISPR knockout screen of CHO cells^29^. This study identified 1980 genes out of the 15,028 targeted that negatively affect cell proliferation and are therefore deemed essential. The iCHO2291 model contains 2291 functionally modelled genes representing CHO cell metabolism, 392 of which are essential according to ref. ^29^. Applying *f* to all reactions within the model containing GPR rules (4182) classified 3296 reactions as non-essential and 886 as essential. These reaction essentiality values allowed us to generate node labels for 3310 nodes within the MFG, with 2498 being non-essential (0) and 812 essential (1).

### Model architecture and training

FluxGAT (Fig. 1) is a graph attention network (GAT) that learns reaction embeddings from the MFG and its node features to predict experimentally determined essentiality labels. Training FluxGAT using labelled data (i.e., supervised learning) enabled the model to learn biologically meaningful representations to predict essentiality labels. During evaluation, the graph was partitioned into training (80%) and testing (20%) sets, ensuring node features designated for testing were masked during the training phase.

We employed k-fold cross-validation to partition the graph systematically into five distinct folds for training (2648 nodes) and testing (662 nodes), ensuring each fold served as a testing set once. This procedure allowed every reaction in the graph to be part of both the training and testing sets across different iterations, mitigating split-dependent bias (i.e., performance being driven by one favourable train-test partition) and testing the generalisability of the model. FluxGAT contains an initial embedding layer that transforms the sparse one-hot reaction node feature vectors—encoding the reactants and products involved in each reaction—into a lower-dimensional continuous representation, improving parameter efficiency and enabling subsequent message passing over the MFG. The input node features are one-hot reaction vectors encoding the reactants and products involved in each reaction; for example, the hexokinase, D-glucose:ATP (HEX1) reaction in iCHO2291, D-glucose + ATP → D-glucose 6-phosphate + ADP + H^+^, is encoded as a binary vector of length ∣*R*∣ with five non-zero entries at the positions corresponding to D-glucose, ATP, D-glucose 6-phosphate, ADP and the proton, and zeros elsewhere.

To select the optimal model settings for FluxGAT, we performed two small 5 × 5 grid searches (25 configurations per sweep and 50 total), as described in the *Hyperparameter optimisation* section. The sweeps covered hidden channels *C* ∈ {10, 20, 40, 80, 150} versus message-passing layers *L* ∈ {1, 2, 3, 4, 5}, and embedding dimension *d* ∈ {25, 50, 100, 150, 300} versus the same layer range. For each setting, we trained the model on the training folds and selected the configuration that maximised the validation F1 score, which balances precision and recall under class imbalance.

The configuration with the highest mean validation F1 score across folds used 150-dimensional embeddings, two message-passing layers (with two attention heads), and 150 hidden channels. We used two attention heads to capture complementary neighbourhood-weighting patterns without substantially increasing parameter count or overfitting risk on this imbalanced dataset. With two message-passing layers, each reaction embedding aggregates information up to its two-hop neighbourhood in the MFG, in addition to its own node features; deeper models (3–5 layers) did not improve validation performance in our hyperparameter tuning and caused oversmoothing. The resulting node representations were passed to a final linear layer to produce a single logit per reaction, followed by a sigmoid activation function to obtain an essentiality probability, to which we applied a fixed decision threshold of 0.65 in the reported results, chosen as a precision-recall trade-off that favoured higher precision under class imbalance while retaining useful recall. To account for the imbalance between essential and non-essential reactions, we used a class-weighted loss with weights proportional to the inverse class frequencies (2498 non-essential vs. 812 essential reactions). We trained using the AdamW optimiser^30^ with standard regularisation (dropout, weight decay) and early stopping.

### FluxGAT performance

We begin by comparing FluxGAT against three traditional binary classifiers: support vector classifier (SVC), multilayer perceptron (MLP), and random forest (RF). The performance of each model, including accuracy, precision, recall, and F1 score, was averaged across five repetitions, each with a different random seed, of 5-fold cross-validation to ensure robustness and consistency in our evaluation.

A manual feature extraction process was employed to generate node features from the MFG to implement the traditional binary classifiers. This process involved creating vectors for each node, composed of the incoming and outgoing edge weights associated with each node, effectively capturing the flow of metabolites into and out of each metabolic reaction’s one-hop neighbourhoods. For example, a reaction with three incoming edges from upstream producers and two outgoing edges to downstream consumers is represented by a five-element vector containing the corresponding MFG edge weights, padded with zeros to a fixed length across reactions.

**Fig. 2:**
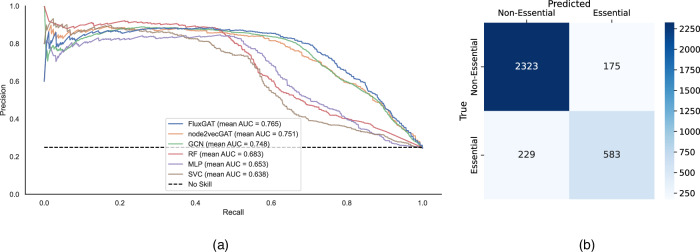
Reaction-level classification performance of FluxGAT compared with baseline classifiers on the iCHO2291 MFG. **a** Precision-recall (PR) curves for FluxGAT, node2vecGAT, GCN and three traditional binary classifiers, averaged across 25 evaluations (5 repetitions of 5-fold cross-validation). The dashed line represents the precision (0.249) of the no-skill classifier given the class imbalance. **b** Confusion matrix showing the average classification performance of FluxGAT on the 3310 experimentally determined reaction essentiality labels.

Figure 2a presents the precision-recall (PR) curves for FluxGAT and the baseline classifiers, averaged across the 25 evaluations (5 repetitions of 5 folds). This choice of PR curves was due to the class imbalance in the MFG, as they provide a more informative performance measure under such conditions compared to traditional receiver operating characteristic (ROC) curves. FluxGAT achieves the highest precision-recall area under the curve (PR-AUC) at 0.765, a significant improvement over the *no-skill* classifier, which has a precision of 0.249, indicative of the class imbalance.

The corresponding confusion matrix for FluxGAT at the fixed decision threshold of 0.65 is shown in Fig. 2b, summarising the distribution of true and false positives/negatives across the 3310 labelled reactions.

In addition to comparing FluxGAT with traditional binary classifiers, we further evaluated its performance against the same GAT architecture and hyperparameters but implemented with *node2vec* embeddings^31^. This comparison allows us to determine the impact of the custom embeddings on FluxGAT’s performance. The node2vecGAT model serves as a GNN benchmark in which initial node embeddings are generated by node2vec from the MFG topology alone (using random-walk-based representations of graph structure). This contrasts with FluxGAT, whose initial node features encode the chemistry of each reaction as a one-hot vector over its reactants and products (Section ‘Node feature generation’). Comparing the two, therefore isolates the contribution of chemistry-aware input features, while keeping the GAT architecture and message passing fixed. Lastly, we evaluated the performance of our framework by substituting a graph convolutional network (GCN) for the GAT architecture. Both GNN benchmarks surpassed the traditional binary classifiers, underscoring the efficacy of learning node representations through message passing over the MFG, rather than relying on manual feature extraction to encode network topology. Notably, FluxGAT, with its incorporation of attention mechanisms and custom node features, achieved a significant enhancement in Precision-Recall Area Under the Curve (PR-AUC). Table 1 compares FluxGAT’s performance to baseline models.

**Table 1:** Comparative performance metrics providing a detailed overview of the mean and standard deviation for test accuracy, precision, recall, and F1 score, aggregated over 25 evaluations (comprising five repetitions of 5-fold cross-validation) for each binary classifier

Method	Accuracy	Precision	Recall	F1 Score
FluxGAT	**0.871** ± **0.012**	0.769 ± 0.030	**0.718** ± **0.037**	**0.743** ± **0.023**
node2vecGAT	0.864 ± 0.014	0.741 ± 0.040	0.693 ± 0.038	0.714 ± 0.023
GCN	0.870 ± 0.013	0.754 ± 0.030	0.699 ± 0.032	0.725 ± 0.030
RF	0.848 ± 0.010	0.786 ± 0.040	0.520 ± 0.041	0.625 ± 0.039
MLP	0.854 ± 0.011	0.813 ± 0.040	0.528 ± 0.028	0.639 ± 0.027
SVC	0.827 ± 0.010	**0.843** ± **0.050**	0.366 ± 0.027	0.509 ± 0.025

Finally, to isolate the contribution of the flux-derived edge weights, we evaluated the topology-only ablation described in the *Topology-only ablation* subsection. Results are reported in Supplementary Table S1.

### Comparison with flux balance analysis

This section details converting FluxGAT’s binary reaction predictions into gene essentiality labels for comparison with FBA. To generate the FBA baseline, we performed single-gene deletion analysis using the model’s default biomass reaction as the optimisation objective. To achieve this, we first computed the wild-type optimal growth rate, *μ*_WT_, and then re-optimised after each gene knockout to obtain ${\mu }_{g}^{KO}$. A gene was classified as essential if the knockout growth ratio ${\mu }_{g}^{KO}/{\mu }_{WT}$ was below a viability threshold *τ*, and non-essential otherwise. We used the standard threshold *τ* = 0.01 (1% of wild-type growth), which is commonly used in GSMM essentiality analyses^32,33^, and did not tune the objective function or threshold to maximise agreement with the experimental labels. A sensitivity analysis of *τ* was performed (Supplementary Section S7), which showed that FBA’s single-deletion growth ratios are strongly bimodal and therefore contain only a narrow band of non-degenerate operating points, with FluxGAT achieving a higher F1 score than FBA in all cases.

To then perform the conversion of FluxGAT reaction labels, we applied the inverse of *f* (Eq. (8)) to the reactions where GPR rules allow a unique inverse mapping. Specifically, this includes reactions with one-to-one mappings to genes, essential reactions governed only by OR operators, and non-essential reactions exclusively involving AND operators in their GPR rules. Out of the 2085 genes present in the MFG, 752 are involved in uniquely invertible mappings.

Importantly, since a single gene may participate in multiple reactions, for gene-level evaluation, we therefore restrict to genes whose reaction-level essentiality labels are consistent across all uniquely invertible reactions in which the gene participates (i.e. all such reactions map to the same gene label). Genes that would require an ambiguous ‘any-reaction’ rule (essential if *any* associated reaction is predicted essential) are excluded from the gene-level comparison to ensure a well-defined mapping and a fair, like-for-like comparison to FBA.

**Fig. 3:**
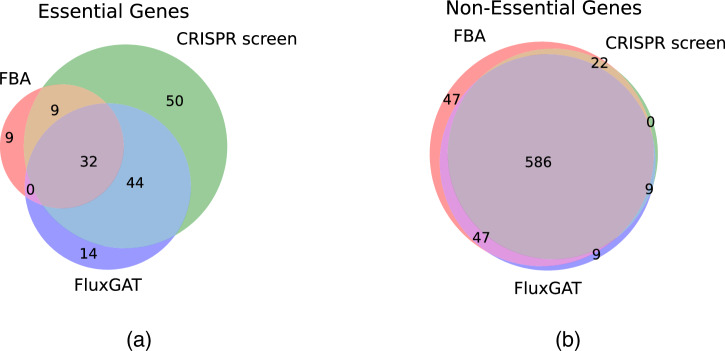
Comparison of iCHO2291 gene essentiality predictions with experimental CRISPR screen, for genes involved in MFG reactions with a unique inverse mapping. **a** Essential gene predictions by FBA and FluxGAT. **b** Non-essential gene predictions by the same methods.

Applying this inverse mapping to reactions labelled by FluxGAT, we can compare the essentiality classification of genes to those made by FBA (also across five repetitions). Figure 3 shows the agreement between FluxGAT and FBA gene essentiality labels with those determined experimentally by a genome-wide CRISPR screen^29^. FluxGAT can identify 32 of the 41 essential genes correctly labelled by FBA and an additional 44 genes missed by FBA (Fig. 3a). However, this comes at the cost of a further 14 false-positive predictions. FluxGAT can also identify nearly all non-essential genes correctly labelled by FBA but with only 56 false negatives instead of 94 (Fig. 3b).

As observed by Strain et al.^21^, the iCHO2291 GSMM displays very high specificity, rarely classifying a non-essential gene as essential, but shows lower sensitivity, failing to capture all essential genes when using FBA. We also observed this in our analysis when classifying the 752 genes in the MFG, whereby FBA displays a specificity of 0.985 but a sensitivity of 0.304. FluxGAT shows similar specificity, with 0.977, but an increased sensitivity of 0.576, capturing nearly double the number of essential genes as FBA.

### Extension to the Mouse1 genome-scale metabolic model

To assess whether FluxGAT generalises beyond CHO cells, we applied the same workflow to Mouse1, the generic mouse GSMM implemented in the Mouse-GEM repository^34^. We first performed flux sampling under the standard growth medium, using the same ACHR algorithm as with iCHO2291. This produced a mean flux vector with 12,359 non-zero reaction fluxes. Using this vector and the stoichiometric matrix for Mouse1, we constructed an MFG with 11,924 reaction nodes and 2,813,137 weighted edges.

We then obtained gene essentiality labels from the Online GEne Essentiality database (OGEE), which compiles data from many mouse gene knockout studies^35^. In line with Wang et al.^34^, we grouped OGEE genes labelled as ‘conditional’, essential in some but not all of the tested conditions, with essential genes. The Mouse1 model contains 2846 genes, of which 1575 have OGEE annotations after mapping via gene symbols and identifiers. Applying the function *f* from Eq. 8 to reactions with GPR rules classified 3566 reactions as essential and 1571 as non-essential. As in the CHO analysis, we then mapped these reaction labels onto the MFG nodes. Because the MFG representation unfolds reversible reactions into separate forward and reverse nodes (as described in the *Graph construction* section), this resulted in labels for 10,274 nodes in the MFG, of which 69.4% were essential.

We re-trained FluxGAT on the Mouse1 MFG using the same architecture and training procedure as for iCHO2291 (as described in the *Model architecture and training* section). We again used 5-fold cross-validation, repeated five times, and computed accuracy, precision, recall, and F1 score on the held-out nodes in each fold. Across the 25 evaluations, FluxGAT achieved a mean accuracy of 0.675, precision of 0.934, recall of 0.577, and F1 score of 0.713 on the reaction labels. These results show that FluxGAT can learn informative flux-sampling-based features in a different mammalian model with a distinct network structure and label source.

For completeness, we also benchmarked FluxGAT against the same baseline classifiers used for iCHO2291 (node2vecGAT, GCN, RF, MLP and SVC) using identical node features and cross-validation splits. The relative ranking of methods on Mouse1 mirrored the CHO results (Supplementary Fig. S6a), with FluxGAT achieving the highest PR-AUC, followed by GCN and node2vecGAT, while feature-based classifiers (RF, MLP, SVC) performed less well. This suggests that the benefit of using flux-informed message passing over manual feature extraction is consistent across both genome-scale models.

We next compared FluxGAT to FBA at the gene level, as in the *Comparison with flux balance analysis* section. We applied the inverse mapping of *f* to the Mouse1 reactions with GPR rules that allow a unique inverse mapping back to genes. This set includes reactions with one gene, essential reactions governed only by OR operators, and non-essential reactions governed only by AND operators. In Mouse1, this yielded 547 genes with uniquely invertible mappings.

**Fig. 4:**
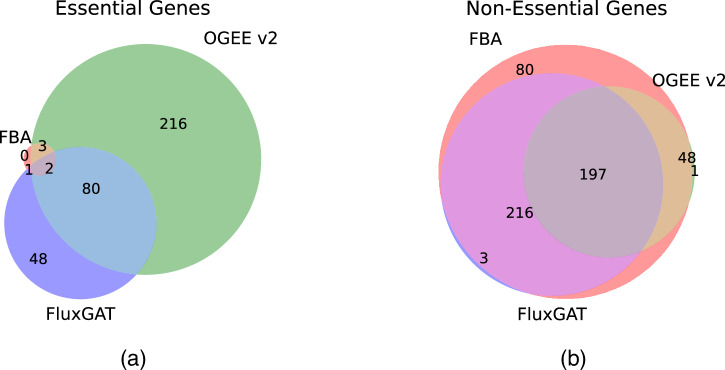
Comparison of Mouse1 gene essentiality predictions with OGEE annotations, for genes involved in MFG reactions with a unique inverse mapping. **a** Essential gene predictions by FBA and FluxGAT. **b** Non-essential gene predictions by the same methods.

For these genes, FBA predicted very few essential genes (Fig. 4a). Using the OGEE labels as ground truth, FBA achieved a sensitivity of 0.0166 and specificity of 0.996 (precision = 0.833, recall = 0.0166). In contrast, FluxGAT reached a sensitivity of 0.272 and specificity of 0.801 (precision = 0.626, recall = 0.272). FluxGAT therefore recovered more than an order of magnitude more experimentally essential genes than FBA on Mouse1, at the cost of a moderate increase in false positives (Fig. 4b). This mirrors our findings in CHO and indicates that the gain in sensitivity provided by FluxGAT is not specific to a single cell line or reconstruction.

### Assessing the practical utility of FluxGAT

The performance metrics stated thus far summarise the predictive accuracy of FluxGAT, but do not show *which* biological functions it recovers beyond objective-based FBA or how its predictions can guide follow-up experiments. To link model performance to practical use, we examined genes for which FluxGAT and FBA disagreed in both iCHO2291 and Mouse1.

For each model, we restricted the comparison to genes with uniquely invertible reaction-to-gene mappings (as per the *Comparison with flux balance analysis* section) and split this set into two groups. First, we defined *recovered essentials* as genes labelled essential in the experimental reference (CRISPR screen for iCHO2291 and OGEE for Mouse1) that FluxGAT predicted as essential but FBA classified as non-essential. Second, we defined *FluxGAT-only positives* as genes predicted essential by FluxGAT but labelled non-essential in the corresponding reference dataset. The recovered-essential group captures cases where FluxGAT increases sensitivity relative to FBA, and the FluxGAT-only group provides a ranked set of candidate context-dependent essentials, while we note that discrepancies can also reflect label noise or differences between experimental and modelling conditions.

#### Pathway enrichment of recovered essentials

First, we sought to answer whether recovered essentials fall into specific biological processes rather than being scattered across the network. To do this, we used g:Profiler (g:GOSt) for functional enrichment analysis with a custom background defined as all genes represented in the corresponding GSMM^36^. In Mouse1, FluxGAT recovered *n* = 80 essential genes that FBA missed (Fig. 4a). These genes are enriched for carbohydrate-derivative and sugar-phosphate metabolic processes, including fructose-6-phosphate and glucose-6-phosphate metabolism, and for glycoprotein metabolism (Supplementary Fig. S8), which indicates that the additional true positives concentrate in central metabolic pathways rather than appearing at random. In iCHO2291, the recovered-essential set (*n* = 44, Fig. 3a) is enriched for de novo IMP and XMP biosynthetic processes (Supplementary Fig. S7), which points to a systematic limitation of objective-based FBA in representing nucleotide biosynthesis demands under the model’s default environmental conditions.

#### External evidence for candidate context-dependent essentials

To further validate FluxGAT, we used evidence that does not depend on the training labels by mapping genes in both models to mouse orthologs and queried organism-level knockout phenotypes from the International Mouse Phenotyping Consortium (IMPC) resource^37^. Across both models, several FluxGAT-only positive genes that FBA missed and that are not labelled essential in the cell-based reference sets have strong organismal viability phenotypes in IMPC, including preweaning lethality for *Slc25a38* and *Mthfd1l* in the CHO analysis, and *Slc25a38* and *Chkb* in Mouse1. These genes sit in biologically plausible parts of the network, for example *Slc25a38* in mitochondrial glycine/haem metabolism and *Mthfd1l* in mitochondrial one-carbon metabolism, which supports targeted validation under appropriate culture conditions. Organismal lethality does not imply cell-line essentiality, so we interpret these phenotypes as independent evidence that these genes are under strong fitness constraint and represent high-priority hypotheses rather than confirmed essentials.

Taken together, these analyses show how FluxGAT can increase sensitivity relative to FBA, and can also be used to identify disagreement sets for generating biologically structured, testable hypotheses. We provide full gene lists, IMPC labels, and enrichment outputs in Supplementary Data 1 (iCHO2291) and Supplementary Data 2 (Mouse1).

## Discussion

In this research, we introduced FluxGAT, a GNN-based approach for predicting metabolic gene essentiality without the observer bias inherent in FBA, the most commonly used method in systems biology. We first applied FluxGAT to the iCHO2291 model of Chinese hamster ovary (CHO) cells and then extended the analysis to Mouse1, the generic mouse GSMM. In both cases, FluxGAT learned from flux sampling distributions and reaction network topology to predict experimental gene essentiality labels.

The core innovation of FluxGAT is the combination of data derived from flux sampling with a graph attention network. This approach allows the model to base its predictions on the structure and use of the metabolic network rather than on predefined cellular objectives. This feature is particularly advantageous for studying mammalian and non-model organisms, where objectives are not well defined, offering an unbiased approach to predicting cellular phenotypes.

Our findings show that FluxGAT surpasses FBA in terms of sensitivity in both CHO and Mouse1, while maintaining high precision and specificity. In the CHO model, FluxGAT recovers nearly twice as many essential genes as FBA among genes with uniquely invertible GPR rules. In Mouse1, FBA predicts very few essential genes when benchmarked against OGEE, whereas FluxGAT increases sensitivity by more than an order of magnitude at a moderate cost in specificity. These results suggest that flux-informed attention-based models can provide more informative gene essentiality predictions than FBA for practical tasks such as target discovery or strain design.

A topology-only ablation clarified where FluxGAT’s predictive signal originates. Replacing flux-derived MFG edge weights with a constant value, while keeping the directed producer-consumer structure of the graph, produced a modest reduction in F1 score for both iCHO2291 and Mouse1 (Supplementary Table S1). It is therefore understood that flux sampling contributes to FluxGAT’s performance in two ways; first by defining which reactions and directions carry feasible flux and enter the graph as active nodes and edges, and second by assigning edge weights that reflect the magnitudes of mass-flow. The first role accounts for most of the predictive signal, with edge-weight magnitudes providing a smaller additional contribution.

Another objective-based alternative to standard FBA is the minimisation of metabolic adjustment (MOMA), which estimates post-perturbation flux states by penalising deviation from the wild-type flux distribution after gene deletion^38^. MOMA may provide a useful additional comparator in future evaluations of FluxGAT, however, in this study, we focused on FBA-derived baselines, since a full MOMA benchmark across both genome-scale models was beyond the computational scope of the present work. More broadly, MOMA remains an optimisation-based approach and therefore does not address the central motivation of this study to reduce reliance on explicitly defined cellular objectives.

FluxGAT also has clear limitations. The current model operates on a static metabolic network and does not include gene-regulatory or signalling interactions, which are known to affect essentiality, particularly in developmental contexts. The training labels are restricted to genes covered by genome-wide screens or curated databases and are therefore incomplete and sometimes noisy. Additionally, FluxGAT is evaluated on single-gene essentiality and because it predicts essentiality at the reaction level before mapping back to genes, it does not capture higher-order genetic interactions such as synthetic lethality, where viability depends on combined perturbations even when no individual reaction is essential on its own. Lastly, FluxGAT carries a higher upfront cost than FBA, requiring around 50 min for flux sampling, 2–3 min for MFG construction and around 10 min for training on our HPC infrastructure, compared with around 30 s for a full FBA essentiality screen (Supplementary Section S9.3). This setup cost is paid once per model and condition, after which gene-by-gene predictions are effectively instantaneous, but the pipeline is less practical when only a single reconstruction needs to be screened or when computational resources are scarce.

Future work will focus on extending FluxGAT to more context-specific models and additional cell types, and on testing its behaviour under different environmental conditions. Another priority is to incorporate multi-layer information, such as gene-regulatory networks or expression data, to improve coverage of non-metabolic genes. We are also interested in studying the attention weights learned by FluxGAT when predicting essentiality. By examining which neighbours and flux patterns the model attends to, we hope to gain insight into the local network features that drive essential or non-essential behaviour.

In conclusion, FluxGAT shows that it is possible to predict gene essentiality directly from genotype and flux patterns, with less reliance on hand-crafted objective functions. Its ability to improve on FBA in two distinct mammalian genome-scale models suggests that this approach can support more general gene essentiality prediction across biological systems. This points to a useful role for deep learning in strengthening in silico tools used in systems biology, with potential impact on personalised medicine and targeted drug development.

## Methods

### Flux sampling

Constraint-based modelling provides a scalable framework for analysing genome-scale metabolic networks without requiring detailed kinetic parameters. For a given network, the relationship between the *m* metabolites and *n* reactions is described by the *m* × *n* stoichiometric matrix *S*. A positive stoichiometric coefficient *S*_*i*,*j*_ indicates that metabolite *i* is produced by reaction *j*, whereas a negative entry indicates that it is consumed. Lower and upper bounds then constrain the allowable rate and directionality of each reaction, while a steady-state assumption enforces mass balance across the network. Under these constraints, the feasible flux space is defined by

1 $$\frac{d{\bf{x}}}{dt}=S{\bf{v}}=0,{{\bf{v}}}^{lb}\le {\bf{v}}\le {{\bf{v}}}^{ub}.$$

**Fig. 5:**
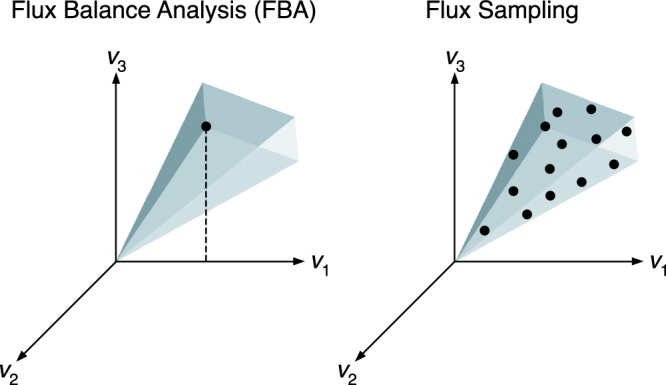
Schematic comparison of flux balance analysis (FBA) and flux sampling. FBA generates a single non-unique solution, and flux sampling generates a probability distribution of feasible solutions of the constrained solution space (blue region) of reaction fluxes v_1_, v_2_, v_3_.

Where **x** is a vector of metabolite concentrations and **v** is a vector of flux rates, constrained by lower and upper bounds for each reaction. FBA introduces an objective function and uses linear programming to find a non-unique flux distribution v^*^, which satisfies the constraints. Flux sampling, on the other hand, aims to uniformly sample the feasible solution space, given the constraints, avoiding the need to specify an objective function. Figure 5 visualises this distinction.

The Hit-and-Run (HR) algorithm is a stochastic Markov chain Monte Carlo (MCMC) method that is widely used for sampling large, genome-scale networks due to its efficiency and convergence behaviour^39^. In this work, an implementation of the artificial centring hit-and-run (ACHR) algorithm, known as *optGpSampler*^40^, was used due to its established application to GSMMs.

In the first iteration (*a* = 0) of the ACHR algorithm, an arbitrary starting point **v**^(0)^ ∈ *P*, is selected within an *N* dimensional convex set *P*, giving an initial centre of $\widehat{{\bf{c}}}={{\bf{v}}}^{(0)}$. The algorithm then generates a chain of samples by iteratively performing the following three steps. First, computing a direction to travel in ***θ***,

2 $${{\boldsymbol{\theta }}}^{(a)}=\frac{{{\bf{v}}}^{(i)}-\widehat{{\bf{c}}}}{| | {{\bf{v}}}^{(a)}-\widehat{{\bf{c}}}| | },\,\,where\,i \sim U\{0,1,\ldots ,a\}.$$

Then, after randomly generating a step size *λ*^(*a*)^, a new sample is calculated **v**^(*a*+1)^ = **v**^(*a*)^ + *λ*^(*a*)^***θ***^(*a*)^. Lastly, updating the artificial centre by setting,

3 $$\widehat{{\bf{c}}}=\frac{a\widehat{{\bf{c}}}+{{\bf{v}}}^{(a)}}{a+1}.$$

This process is repeated until convergence, as confirmed by diagnostic tests, resulting in probability distributions of possible flux values for each reaction. Then, a single flux distribution is calculated by averaging the probability distribution for each reaction to generate an *n*-dimensional flux vector $\overline{{\bf{v}}}$, facilitating downstream analysis, as was done in ref. ^21^.

### Graph construction

There are numerous ways to construct a graphical representation of a given metabolic reaction network using its stoichiometry, however, many fail to capture the directionality of reactions and are dominated by pool metabolites that appear in many reactions, such as water. The MFG was designed to address these common limitations by utilising flux distributions to encode the directionality of metabolic flows whilst mitigating the over-representation of pool metabolites^27^, producing a directed graph. The MFG also exhibits systemic changes to its topological and community structure under environmental and genetic perturbations, revealing redundancies and core structures within a reaction network. It is, therefore, well-suited for highlighting essential components.

An MFG can be constructed from a stoichiometry matrix *S* whereby nodes represent reactions and a directed edge connects two nodes if the source reaction produces a metabolite consumed by the target reaction. As detailed in ref. ^27^, starting with flux vector, obtained either through FBA v^*^, or in our case, averaged across flux sampling iterations $\overline{{\bf{v}}}$, we first unfold the vector into the forward and reverse components of each reaction,

4 $${\bar{{\bf{v}}}}_{2n}=[\begin{array}{l}{\bar{{\bf{v}}}}^{+}\\ {\bar{{\bf{v}}}}^{-}\end{array}]=\frac{1}{2}\left[\begin{array}{l}{\mathrm{abs}}(\bar{{\bf{v}}})+\bar{{\bf{v}}}\\ {\mathrm{abs}}(\bar{{\bf{v}}})-\bar{{\bf{v}}}\end{array}\right].$$

The reaction-wise mean sampled flux was used to define the flux vector $\overline{{\bf{v}}}$ for MFG construction, consistent with prior work using sampled flux summaries for downstream GSMM analyses^21,41^. The sensitivity of this choice to alternative summary statistics is described in the *Sensitivity of MFG construction to the flux summary statistic* subsection.

Then, we can define the modified stoichiometry matrix as,

5 $${{\bf{S}}}_{2n}=[\begin{array}{cc}{\bf{S}} & -{\bf{S}}\end{array}]\left[\begin{array}{cc}{{\bf{I}}}_{n} & 0\\ 0 & \mathrm{diag}({\bf{r}})\end{array}\right],$$

where *r* is an *n*-dimensional Boolean reversibility vector such that *r*_*j*_ = 1 if reaction *j* is reversible and 0 otherwise. Lastly, the adjacency matrix of the MFG is given by,

6 $${\bf{M}}(\overline{{\bf{v}}})={({{\bf{S}}}_{2n}^{+}\overline{{\bf{V}}})}^{T}{{\bf{J}}}_{v}^{\dagger }({{\bf{S}}}_{2n}^{-}\overline{{\bf{V}}}),$$

where $\overline{{\bf{V}}}$ and **J**_*v*_ are defined as $diag({\overline{{\bf{v}}}}_{2n})$ and $diag({{\bf{S}}}_{2n}^{+}{\overline{{\bf{v}}}}_{2n})$ respectively, and † denotes the matrix pseudoinverse. Here, ${{\bf{J}}}_{v}^{\dagger }$ performs a metabolite-wise normalisation that down-weights pool metabolites with large total production flux.

Finally, this gives our producer-consumer relationships,

7 $$\begin{array}{cc}\mathrm{Production}\,: & {{\bf{S}}}_{2n}^{+}=\frac{1}{2}\left(\mathrm{abs}({{\bf{S}}}_{2n})+{{\bf{S}}}_{2n}\right),\\ \mathrm{Consumption}\,: & {{\bf{S}}}_{2n}^{-}=\frac{1}{2}\left(\mathrm{abs}({{\bf{S}}}_{2n})-{{\bf{S}}}_{2n}\right).\end{array}$$

Note that for reversible reactions whose mean sampled flux is non-zero in only one direction, the opposite direction contributes a zero entry in ${\overline{{\bf{v}}}}_{2n}$ and therefore produces zero edge weights in ${\bf{M}}(\overline{{\bf{v}}})$. Such directions correspond to isolated nodes in the resulting graph and are removed during MFG construction. Consequently, the number of MFG nodes is generally smaller than 2*n* and reflects only directions that carry feasible non-zero flux within the sampled distribution.

### Sensitivity of MFG construction to the flux summary statistic

Because flux-sampling distributions can be skewed or multimodal for some reactions, we assessed whether the choice of summary statistic used to aggregate sampled fluxes could materially affect the MFG constructed from the sampling output. In the main pipeline, we use the reaction-wise mean sampled flux to define the flux vector $\overline{{\bf{v}}}$ used for graph construction. To test the robustness of this choice, we additionally computed the reaction-wise median sampled flux for every reaction in both iCHO2291 and Mouse1 from the same ACHR sampling output.

We then compared the mean- and median-derived summaries at two levels. First, we quantified reaction-wise agreement between the two summaries across all reactions in each model. Second, because FluxGAT operates on the topology of the resulting MFG, we reconstructed MFGs from both the mean- and median-derived flux vectors and compared their unweighted graph structure. Specifically, we compared the global overlap of the resulting edge sets and the per-node overlap of local neighbourhoods at one hop and at ≤2 hops. The latter is particularly relevant because the final FluxGAT architecture uses two message-passing layers, so its effective receptive field is determined by the two-hop neighbourhood of each node. The corresponding correlation plots, fan plots and topology-overlap summaries are provided in the Supplementary Information.

### FBA viability threshold sensitivity

To assess the robustness of the FBA baseline used for comparison with FluxGAT, we performed a sensitivity analysis with respect to the viability threshold *τ* used to classify a gene knockout as essential or non-essential. In the main analysis, a gene was classified as essential when the knockout growth ratio ${\mu }_{g}^{KO}/{\mu }_{WT}$ was below *τ* = 0.01, corresponding to 1% of wild-type growth. This threshold was selected a priori as a standard setting in GSMM essentiality analyses and was not tuned to maximise agreement with the experimental labels.

For the sensitivity analysis, we repeated the FBA single-gene deletion procedure across a range of threshold values for each GSMM. At each threshold, we recorded the number of genes classified as essential, the change in essential-gene count relative to the default threshold, and the overlap between the essential-gene set at that threshold and the set obtained at *τ* = 0.01. These calculations were performed on the full set of modelled genes in each reconstruction, rather than only the uniquely invertible subset used for the FluxGAT gene-level benchmarking, in order to characterise the behaviour of the FBA baseline itself. The full threshold-sensitivity summaries are reported in the Supplementary Information.

### Node label generation

Considering that the MFG comprises a set of reactions as nodes rather than a set of genes, we must initially map the gene essentialities onto the corresponding set of reaction essentialities to train and test a classifier. To perform this mapping, the method outlined in ref. ^42^ is utilised, where the authors address the inconsistent treatment of gene protein reaction (GPR) rules by defining a standard approach to handling them. GPR rules describe how genes relate to protein complexes and the reactions they catalyse. They contain Boolean logic operators that allow us to classify reactions as active/inactive using gene expression.

These GPR rules can also be processed to translate gene essentiality into node essentiality labels according to the following function *f*, applied to each reaction,

8 $$f(\oplus ,{g}_{1},\ldots ,{g}_{n})=\left\{\begin{array}{ll}\max ({g}_{1},\ldots ,{g}_{n}) & \mathrm{if}\,\,\mathrm{only}\,\mathrm{ANDs}\\ \min ({g}_{1},\ldots ,{g}_{n}) & \mathrm{if}\,\mathrm{only}\,\mathrm{ORs}\\ \mathrm{Resolve}\,\mathrm{ANDs} & \mathrm{if}\,\mathrm{mixed}\\ \mathrm{then}\,\mathrm{ORs} & \end{array}\right.$$

where,

- ⊕ : Represents the logical operator or the combination of operators within the GPR rule for a given reaction.
- *g*_1_, …, *g*_*n*_: The sequence of gene essentiality values (1 for essential and 0 for non-essential).
- *Resolve ANDs then ORs*: For mixed operators, standard logical precedence rules apply (AND operations are typically evaluated before OR operations unless parentheses indicate otherwise).

### Node feature generation

GNNs exhibit state-of-the-art performance across various network-based tasks, primarily due to their ability to combine representations of network topology with *side information* such as node features. This section outlines the node (reaction) feature generation process to create embeddings that complement the adjacency matrix, *M*.

Our generated node features contain biologically meaningful information for the downstream classification task of essentiality prediction. This approach involved compiling a list of the reactants and products of each reaction, representing additional chemical properties not captured within the MFG. These lists encode each reaction’s starting material and end result in a structured format, emphasising relationships between nodes.

A one-hot encoding was employed to transform node features into a numerical format suitable for a GNN. To achieve this, we let *R* be the set of all reactants and products across reactions, with ∣*R*∣ being its cardinality. For each reaction *i*, a one-hot encoded vector **o**_*i*_ is constructed, where **o**_*i*_ ∈ {0, 1}^∣*R*∣^. For each reactant or product *j* in *R*, the corresponding element in **o**_*i*_, denoted as *o*_*ij*_, is defined as:

9 $${o}_{ij}=\left\{\begin{array}{ll}1 & \mathrm{if}\,\mathrm{reactant}/\mathrm{product}\,j\,\mathrm{is}\,\mathrm{involved}\,\mathrm{in}\,\mathrm{reaction}\,i,\\ 0 & \mathrm{otherwise}.\end{array}\right.$$

### Graph representation learning

We use a graph neural network (GNN) model to perform representation learning on the MFG due to its ability to combine features of individual reactions with the interaction patterns defining the reaction network topology. Specifically, we employ a graph attention network (GAT)^43^, which integrates self-attention mechanisms, allowing the model to weight neighbouring nodes based on their relevance to a given reaction in the context of the learning task.

Formally, GNNs contain *neural message passing* layers where vector messages are exchanged between nodes and updated using neural networks. In each message-passing layer, the hidden embedding ${{\bf{h}}}_{u}^{(i)}$ for node $u\in {\mathcal{V}}$ is updated by aggregating information from its neighbourhood ${\mathcal{N}}(u)$:

10 $$\begin{array}{ll}{{\bf{h}}}_{u}^{(i+1)} & ={\mathrm{UPDATE}}^{(i)}({{\bf{h}}}_{u}^{(i)},\\ & {\mathrm{AGGREGATE}}^{(i)}(\{{{\bf{h}}}_{v}^{(i)}\,\forall v\in {\mathcal{N}}(u)\}))\\ & ={\mathrm{UPDATE}}^{(i)}({{\bf{h}}}_{u}^{(i)},{{\bf{m}}}_{{\mathcal{N}}(u)}^{(i)})\end{array}$$

where UPDATE and AGGREGATE are arbitrary differentiable functions and ${{\bf{m}}}_{{\mathcal{N}}(u)}$ is the ‘message’ that is aggregated from *u*’s one-hop graph neighbourhood ${\mathcal{N}}(u)$ at iteration *i*. Here, we use a modified version of the original GAT, assigning a learnable attention weight to each neighbour based on node and edge features. This attention weight determines the *importance* of nodes during the aggregation step, with each message defined as

11 $${{\bf{m}}}_{{\mathcal{N}}(u)}=\mathop{\sum }\limits_{v\in {\mathcal{N}}(u)}{\alpha }_{u,v}{{\bf{h}}}_{v},$$

where *α*_*u*,*v*_ denotes the attention given to neighbour $v\in {\mathcal{N}}(u)$ during aggregation at node *u*. The attention weights are calculated based on the importance of the features of the source node, the target node, and the edge, as follows,

12 $${\alpha }_{u,v}=\frac{\exp ({{\bf{a}}}^{\top }[{\bf{W}}{{\bf{h}}}_{u}\oplus {\bf{W}}{{\bf{h}}}_{v}\oplus {\bf{W}}{{\bf{e}}}_{u,v}])}{{\sum }_{{v}^{{\prime} }\in {\mathcal{N}}(u)}\exp ({{\bf{a}}}^{\top }[{\bf{W}}{{\bf{h}}}_{u}\oplus {\bf{W}}{{\bf{h}}}_{{v}^{{\prime} }}\oplus {\bf{W}}{{\bf{e}}}_{u,{v}^{{\prime} }}])},$$

where **a** is a trainable attention vector, **W** are trainable matrices unique to each feature, and ⊕ denotes the concatenation operation. Multi-dimensional edge features between nodes *u* and *v* are represented by **e**_*u*,*v*_, which, in our case, are equal to scalar edge weights of the MFG representing chemical mass flow between reactions.

The initial node embeddings at *i* = 0, ${{\bf{h}}}_{u}^{(0)}$, are set to the one-hot encodings detailed in the *Node feature generation* section. After running *N* iterations of the GNN message passing, we can use the output of the final layer to define the embeddings for each node, i.e.,

13 $${{\bf{z}}}_{u}={{\bf{h}}}_{u}^{(N)},\forall u\in {\mathcal{V}}.$$

Then, to perform node classification, **z**_*u*_ is passed through a dense neural layer to generate a single logit *z*_*u*_, which is passed through a sigmoid activation function to determine the binary class (essentiality) for node (reaction) *u*.

### Hyperparameter optimisation

We selected the FluxGAT architecture using a grid-search procedure over a small set of model hyperparameters controlling representation size and message-passing depth. The search considered the dimension of the initial embedding layer used to compress the sparse one-hot reaction feature vectors, the number of hidden channels in the graph attention layers, and the number of message-passing layers. For each hyperparameter configuration, the model was trained on the training folds only, and performance was evaluated on the corresponding validation folds.

Model selection was based on the validation F1 score, which provides a balanced summary of precision and recall given the class imbalance. The final configuration was chosen as the setting that maximised the mean validation F1 score across folds. This procedure was used to select the architecture reported in the results, and the full search space together with the corresponding heatmaps are provided in the Supplementary Information. By tuning model depth explicitly, this search also allowed us to assess the trade-off between enlarging the effective receptive field and the risk of oversmoothing on large metabolic graphs.

### Topology-only ablation

To isolate the contribution of the flux-derived edge weights from that of graph topology alone, we performed a topology-only ablation of the MFG. In this analysis, we preserved the directed producer-consumer structure of the graph but replaced all non-zero edge weights with a constant value of 1. This removes information about the magnitude of mass flow between reactions while retaining the same neighbourhood connectivity and directionality.

The ablation model was trained and evaluated using the same node features, architecture, cross-validation splits and optimisation procedure as the default FluxGAT model. Any performance difference between the weighted and unweighted versions can therefore be attributed to the removal of flux-derived edge-weight information rather than to changes in the underlying graph topology or training protocol. The resulting reaction-level performance metrics for both iCHO2291 and Mouse1 are reported in the Supplementary Information.

## Supplementary information

Supplementary Information

Supplementary Data 1

Supplementary Data 2

## Data Availability

Experimentally determined CHO gene essentiality used for ground-truth labels was taken from the virus-free genome-wide CRISPR screen reported by Xiong et al.^29^. Mouse gene essentiality annotations were obtained from the Online Gene Essentiality database (OGEE) v2^35^. Gene essentiality predictions for FBA and FluxGAT using both the iCHO2291 and Mouse1 models can be found in the Supplementary Data files, alongside the ground truth labels from ^29^ and ^35^.

## Code availability

All code to run flux sampling, construct the mass-flow graphs (MFGs), and train/evaluate FluxGAT is openly available at: https://github.com/kierensharma/FluxGAT. This version of the code has been modified to allow execution on personal computers, although users should note that this may significantly increase processing time compared to running the code on an HPC cluster.
