# Supplementary material for: Flux sampling and graph neural networks for improved gene essentiality prediction in mammalian genome-scale metabolic models
Source: NPJ Syst Biol Appl. 2026 May 8;12:107. doi: 10.1038/s41540-026-00738-8 (PMC13369363; doi:10.1038/s41540-026-00738-8)
Supplement: Supplementary file 1 — Supplementary Information [file 41540_2026_738_MOESM1_ESM.pdf]

Supplementary Information

FluxGAT: Integrating Flux Sampling with Graph Neural Networks for Unbiased Gene Essentiality Classification

S1 Sensitivity of MFG construction to the flux summary statistic

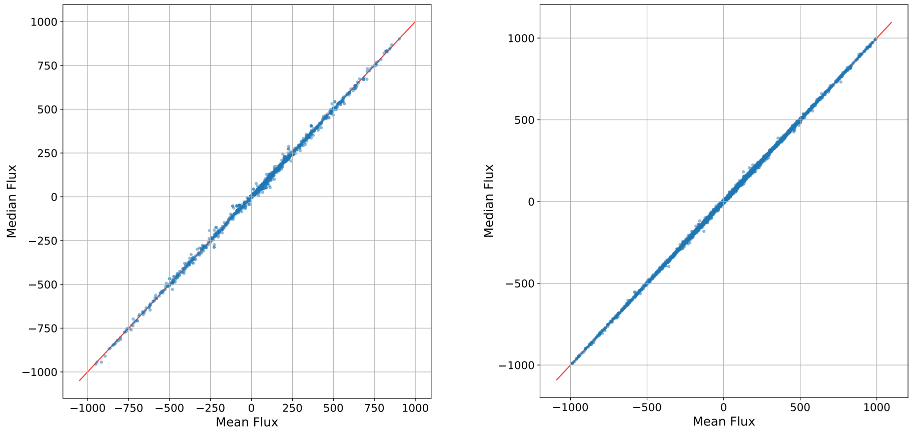

(a) (b)

Supplementary Figure S1: Reaction-wise mean versus median flux for (a) iCHO2291 and (b) Mouse1, computed across 50,000 flux-sampling iterations. In both models, mean and median summaries are nearly perfectly linearly related (iCHO2291: Pearson $R = 0.99984$ and Mouse1: Pearson $R = 0.99927$), indicating that the central tendency of sampled reaction fluxes is highly stable to the choice of summary statistic.

Across both models, reaction-wise mean and median sampled fluxes were nearly perfectly correlated (Supplementary Figure S1). For iCHO2291, the Pearson correlation was $R = 0.99984$, and 62.06% of reactions had skewness scores between -0.5 and 0.5. For Mouse1, the corresponding Pearson correlation was $R = 0.99927$, with 59.67% of reactions in the same skewness range.

At the graph level, mean- and median-derived MFGs were also near-identical. For iCHO2291, the global edge-set Jaccard similarity was 0.99645, with extremely high per-node overlap in both 1-hop and $\leq$2-hop neighbourhoods. Mouse1 showed the same qualitative behaviour, with only marginal differences in non-zero edge counts, global edge-set Jaccard similarity of approximately 0.996, and very high neighbourhood overlap across nodes. These results support the conclusion that

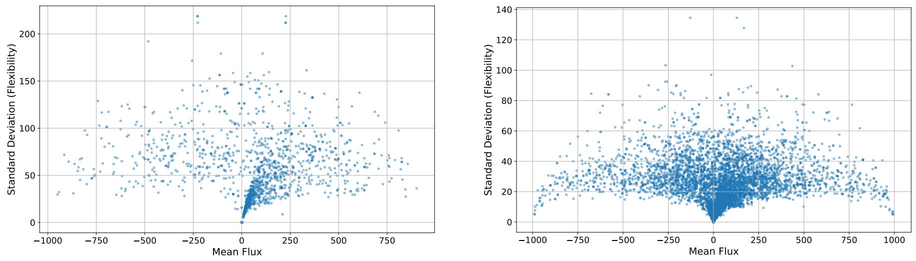

(a) (b)

Supplementary Figure S2: Fan plots showing relationship between reaction flux mean and standard deviation across sampling iterations for (a) iCHO2291 and (b) Mouse1. In both models, high-flux reactions tend to exhibit comparatively low variability, whereas the highest-variance reactions are enriched for transport and exchange processes, consistent with biologically meaningful flexibility in the sampled solution space rather than sampling artefacts.

replacing the mean with the median does not materially change the graph context available to the two-layer FluxGAT model.

As an additional diagnostic, Supplementary Figure S2 shows the relationship between mean flux magnitude and variability. In both models, central high-flux reactions (including ATP synthase and glycolysis-associated reactions) exhibit comparatively low variance, whereas the most variable reactions are dominated by transport and exchange processes (include lactate, alanine and glutamine exchange in iCHO2291, and transport reactions such as `RND213` and `PIt2r` in Mouse1), supporting the interpretation that the sampled flux summaries reflect structured biological behaviour rather than sampling artefacts.

S2 Mass flow graphs (MFGs)

Supplementary Figure S3 provides visualisations of the mass flow graphs (MFGs) constructed from the flux-sampling data in iCHO2291 and Mouse1. These plots help illustrate the overall scale and connectivity of the directed producer-consumer graphs used as the input structure for FluxGAT in each model.

S3 Hyperparameter optimisation

Supplementary Figure S4 summarises the grid-search results used to select the FluxGAT architecture. Across the tested configurations, the best-performing model used 150-dimensional embeddings, 150 hidden channels, and two message-passing layers. The heatmaps also show that performance decreased for deeper models, consistent with oversmoothing on large metabolic graphs and supporting the use of a two-layer architecture in the main analyses.

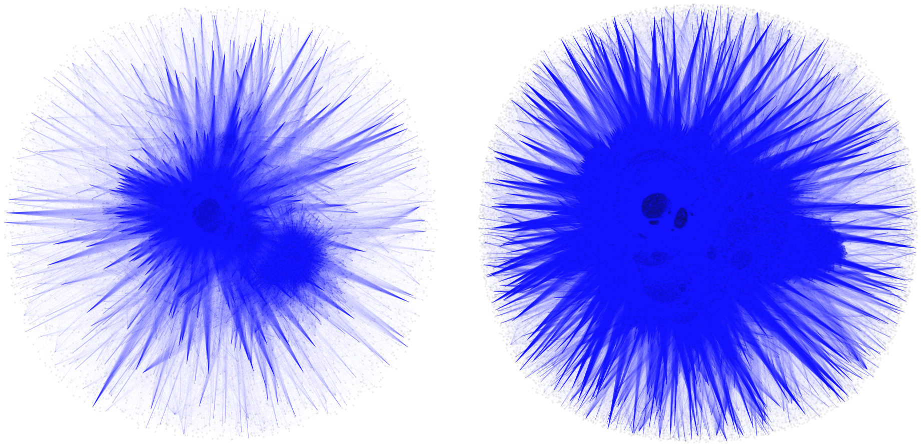

(a) (b)

Supplementary Figure S3: Mass flow graph (MFG) representations of the metabolic reaction networks constructed from flux sampling data. (a) CHO cell model iCHO2291, containing 4,733 reaction nodes and 335,024 edges. (b) Mouse1, containing 11,924 reaction nodes and 2,813,137 edges.

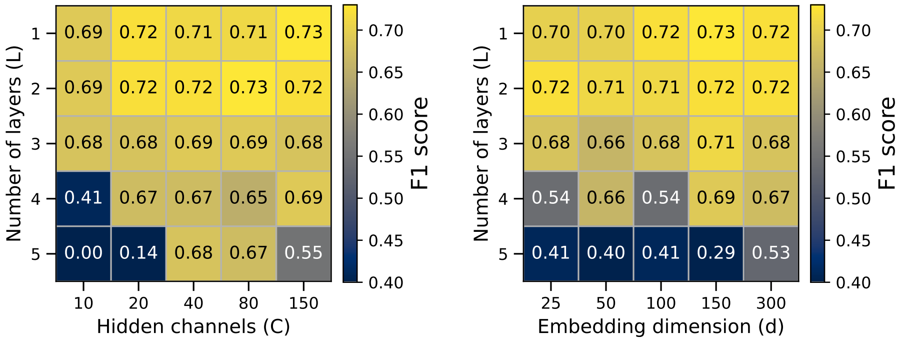

(a) (b)

Supplementary Figure S4: Hyperparameter sweep results (F1 score) across model depth and (a) hidden channels or (b) embedding dimension.

S4 Node embedding space

For an exploration of the feature transformations enabled by FluxGAT, Supplementary Figure S5 illustrates the evolution from initial input features to learned embeddings, highlighting the model's effectiveness in distinguishing between essential and non-essential nodes. Future work will focus on understanding the features that drive this transformation, aiming to identify the specific attributes and interactions FluxGAT leverages to classify essentiality.

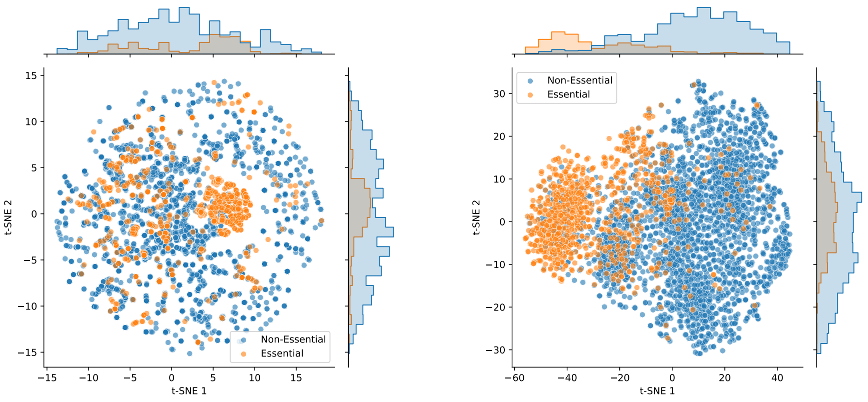

(a) (b)

Supplementary Figure S5: t-SNE visualisations contrasting the structure of input features and learned node embeddings. (a) the initial input features for each reaction, essential (orange) and non-essential (blue), with marginal histograms depicting the distribution of t-SNE components for each class. (b) t-SNE reduced high-dimensional embeddings generated by FluxGAT. Both plots used a perplexity of 30.

S5 Topology-only ablation

Removing flux-derived edge weights while preserving the directed MFG topology caused only a modest reduction in performance in both models (Supplementary Table S1). This indicates that network topology and neighbourhood context provide most of the predictive signal available to FluxGAT, while flux-derived edge weights contribute an additional but smaller improvement.

Supplementary Table S1: FluxGAT performance with flux-weighted MFG edges (default) versus an unweighted-edge ablation (all edge weights set to 1, with directed topology preserved). Metrics are averaged across 5 repeats of 5-fold cross-validation. Δ denotes (weighted $-$ unweighted).

Model	Edge weights	Accuracy	Precision	Recall	F1
iCHO2291	Flux-weighted (default)	0.871	0.769	0.718	0.743
	All weights = 1 (ablation)	0.858	0.706	0.720	0.713
	Δ (weighted $-$ ablation)	+0.013	+0.063	-0.002	+0.03
Mouse1	Flux-weighted (default)	0.675	0.934	0.577	0.713
	All weights = 1 (ablation)	0.669	0.933	0.570	0.707
	Δ (weighted $-$ ablation)	+0.006	+0.001	+0.007	+0.006

S6 Mouse1 reaction-level benchmarking

Supplementary Figure S6a shows the reaction-level benchmarking results for Mouse1. As in iCHO2291, FluxGAT achieved the highest PR-AUC among the evaluated models, and the corresponding confusion matrix indicates strong precision with improved recall relative to the baseline classifiers. These results support the consistency of FluxGAT's reaction-level performance across the two mammalian GSMMs analysed in this study.

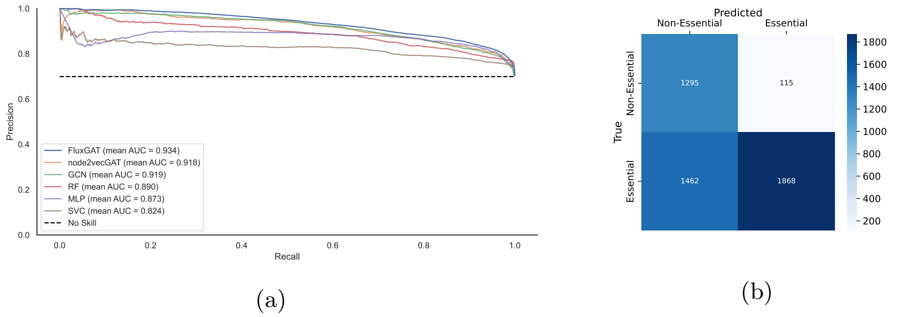

Supplementary Figure S6: (a) Precision-recall (PR) curves for FluxGAT, node2vecGAT, GCN and three standard binary classifiers on the Mouse1 MFG, averaged across 25 evaluations (5 repetitions of 5-fold cross-validation). The dashed line represents the precision (0.706) of the no-skill classifier given the class imbalance. (b) Confusion matrix showing the average classification performance of FluxGAT on the 4,740 experimentally determined reaction essentiality labels.

S7 FBA viability threshold sensitivity analysis

We assessed the sensitivity of FBA essentiality classifications to the viability threshold τ on the uniquely invertible gene subsets used for gene-level comparison with FluxGAT (752 genes in iCHO2291 and 547 genes in Mouse1). At each τ, we recomputed precision, recall, F1 score and specificity, together with the predicted essential-gene count and the Jaccard similarity of the essential-gene set relative to the default $\tau = 0.01$.

In iCHO2291 (Supplementary Table S2), classifications were stable for $\tau \in [0.010, 0.020]$ and shifted slightly for $\tau \in [0.025, 0.030]$, before collapsing to a degenerate regime at $\tau \geq 0.035$ in which all genes are classified as essential. In Mouse1 (Supplementary Table S3), classifications were invariant across the entire tested range $\tau \in [0.010, 0.050]$. This limited operating range reflects the structure of FBA essentiality under a fixed biomass objective, whereby single-deletion growth ratios $\mu_g^{\mathrm{KO}}/\mu_{\mathrm{WT}}$ are strongly bimodal, with most deletions retaining an alternate optimum at near-wild-type growth and a small set dropping sharply towards 0, leaving few intermediate values.

FBA's best non-degenerate F1 in iCHO2291 (0.482 at $\tau \in [0.025, 0.030]$) marginally exceeds its F1 at the default $\tau = 0.01$ (0.443). FluxGAT's gene-level F1, derived from the sensitivity and specificity values reported in the *Comparison with flux balance analysis* section, is approximately 0.69 in iCHO2291 and 0.38 in Mouse1, exceeding FBA's F1 at every non-degenerate τ tested.

Supplementary Table S2: Viability threshold sensitivity for FBA essentiality classifications on the 752-gene uniquely invertible subset in iCHO2291.

Threshold τ	Predicted essential	Precision	Recall	F1	Specificity	Jaccard vs 0.01
0.010	50	0.820	0.304	0.443	0.985	1.000
0.015	50	0.820	0.304	0.443	0.985	1.000
0.020	50	0.820	0.304	0.443	0.985	1.000
0.025	56	0.821	0.341	0.482	0.984	0.893
0.030	56	0.821	0.341	0.482	0.984	0.893
≥ 0.035	752	0.180	1.000	0.304	0.000	0.066

Supplementary Table S3: Viability threshold sensitivity for FBA essentiality classifications on the 547-gene uniquely invertible subset in Mouse1.

Threshold τ	Predicted essential	Precision	Recall	F1	Specificity	Jaccard vs 0.01
0.010-0.050	6	0.833	0.017	0.033	0.996	1.000

S8 GO and reactome enrichment

Supplementary Figures S7 and S8 show the enrichment profiles of the recovered-essential gene sets identified from the FluxGAT-FBA disagreement analysis in iCHO2291 and Mouse1, respectively. In both models, the enriched terms indicate that FluxGAT's additional true positives cluster in coherent biological processes rather than appearing as isolated genes.

S9 Computational resources

S9.1 Hardware specifications

Simulations were conducted using a high-performance computing (HPC) cluster, which is equipped with the following node types:

- Lenovo nx360 m5 compute nodes, each featuring two Intel E5-2680 v4 (Broadwell) CPUs with 14 cores at 2.4 GHz, and 128 GiB of RAM.
- High-memory nodes, each with 512 GiB of RAM.
- GPU-equipped nodes, each housing two graphics processing units.

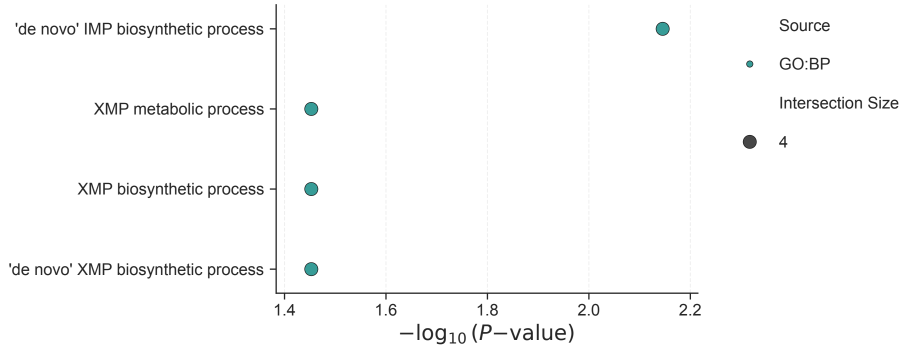

Supplementary Figure S7: Functional enrichment results for the iCHO2291 recovered-essential (FluxGAT true positives missed by FBA) gene set ($n = 44$), using all genes modelled in iCHO2291 as background.

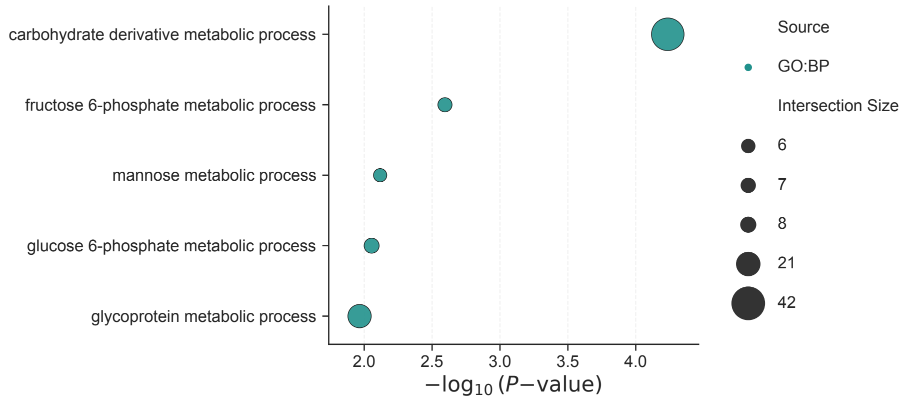

Supplementary Figure S8: Functional enrichment results for the Mouse1 recovered-essential (FluxGAT true positives missed by FBA) gene set ($n = 80$), using all genes modelled in Mouse1 as background.

For the experiments conducted, every node type was strategically utilised, based on the specific requirements of the job and the experiment phase in question. High-memory nodes were specifically employed for flux sampling of the iCHO2291 GSMM, whereas GPU nodes facilitated both the construction of MFGs and the training/evaluation of FluxGAT along with other GNN models. All remaining tasks were efficiently executed on the standard compute nodes.

S9.2 Software and libraries

- Python 3.9
- COBRApy 0.29.0

- PyTorch 2.1.1
- PyTorch Geometric 2.4.0
- NetworkX 2.8.8
- node2vec 0.4.6

S9.3 Approximate runtime comparison

We report approximate runtimes measured on the HPC infrastructure described in Section S9.1. These values are order-of-magnitude estimates and depend on the model, solver configuration and available hardware. Under our settings, using CPLEX for constraint-based optimisation, a full FBA essentiality classification took around 30 seconds and the corresponding pFBA run took around 4 hours (producing identical classifications to FBA under the evaluated settings). MOMA took around 100 seconds per single-gene knockout, making full knockout screening prohibitively slow at the genome scale. The FluxGAT pipeline required around 50 minutes for flux sampling (50,000 iterations), 2-3 minutes for MFG construction from the sampled flux vector, and around 10 minutes for 5-fold cross-validation training. Once the MFG and trained model are available, gene-by-gene essentiality predictions at inference are effectively instantaneous, since they require only a forward pass through the GAT followed by the GPR inverse mapping.
